# Use of Extracorporeal Membrane Oxygenation in Patients with Refractory Cardiac Arrest due to Severe Persistent Hypothermia: About 2 Case Reports and a Review of the Literature

**DOI:** 10.1155/2021/5538904

**Published:** 2021-11-05

**Authors:** Rachid Attou, Sébastien Redant, Thierry Preseau, Kevin Mottart, Louis Chebli, Patrick M. Honore, David De Bels, Andrea Gallerani

**Affiliations:** ^1^Department of Intensive Care Medicine, Brugmann University Hospital, Brussels, Belgium; ^2^Emergency Department, Brugmann University Hospital, Brussels, Belgium; ^3^Department of Vascular Surgery, Brugmann University Hospital, Brussels, Belgium

## Abstract

We report the cases of two patients experiencing persistent severe hypothermia. They were 45 and 30 years old and had a witnessed cardiac arrest managed with mechanized cardiopulmonary resuscitation (CPR) for 4 and 2.5 hours, respectively. Extracorporeal membrane oxygenation was used in both patients who fully recovered without any neurological sequelae. These two cases illustrate the important role of extracorporeal CPR (eCPR) in persistent severe hypothermia leading to cardiac arrest.

## 1. Introduction

Hypothermia is defined as a core temperature below 35°C. Management of severe hypothermia associates rehydration and rewarming after having retrieved the patient from his cold environment [1]. Rehydration usually consists in perfusion of warmed (42°C) isotonic fluids. Combinations of rewarming also include forced warm air, warm infusions, forced peritoneal lavage, or hemofiltration [2]. More recently, venoarterial extracorporeal membrane oxygenation (ECMO) has been the method of choice for in-hospital rewarming in severe hypothermia.

Cardiac arrest is frequent in severe hypothermia. Observational studies suggest that extracorporeal cardiopulmonary resuscitation can be associated with improved survival in very selected patients experiencing cardiac arrest even though randomized controlled trials are lacking [3, 4]. Out-of-hospital cardiac arrests are always associated with less favorable outcomes [5]. We would like to report two young patients with severe hypothermia and refractory long-lasting cardiac arrest treated by extracorporeal membrane oxygenation technique.

## 2. Cases Reports

The first patient, a 45-year-old Caucasian male, was found unconscious in a park this winter. Emergency medical services started cardiopulmonary resuscitation (CPR) on arrival. First rhythm was ventricular fibrillation. The patient received 4 inefficient defibrillation attempts and received a total of 4 mg of epinephrine after asystole appeared. Core temperature was 22.7°C on a low-reading thermometer. Main medical history was chronic alcohol abuse. The patient was homeless. On hospital arrival, the patient was put under a Lund University Cardiac Arrest System (LUCAS®) while awaiting the ECMO team. Admission arterial gas analysis showed a severe mixed acidosis associated with hypoxia: pH 6.98, PaCO_2_ 57, and PaO_2_ 49. Laboratory testing showed anemia with Hb 9.8 g/dL (13-18), a thrombocytopenia at 65000/mm^3^ (150-440 000), and leucopenia with a total white cell count of 2470/mm^3^ (3.5-11). Coagulation disorders were also present. Admission ethanol was 2.4 g/L. No liver cytolysis or cholestasis was seen. The patient was transferred to the Intensive Care Unit. A venoarterial extracorporeal membrane oxygenation system was installed 110 minutes after hospital arrival (flow 1.8 L/min/m^2^, FiO_2_ 100% with femorofemoral approach including a 25 F venous catheter and a 19 F arterial catheter. The total time of CPR was 4.0 hours. Unfractionated heparin was given to keep anti-Xa between 0.5 and 0.7 IU/mL). At 33°C, a new defibrillation resulted in the appearance of a regular sinus rhythm. The patient was under mechanical ventilation as from admission on a volume control basis with a tidal volume of 420 mL, 20 times per minute with at first an FIO_2_ of 100% then after VA ECMO and return to sinus rhythm after correction of hypothermia; the FiO_2_ was decreased to 40% in order to maintain SpO_2_ at 95%. Pulmonary infection with H. influenzae led to antimicrobial therapy by amoxiclav for 5 days. Seeing the total cardiovascular recuperation, ECMO was weaned on day 4 by the surgical team. Thorough neurological examination was done on days 3, 5, and 6 with good results. The patient was extubated on day 6. He left the ICU on day 7 and the hospital on day 14 without any neurological sequelae.

**Figure 1:**
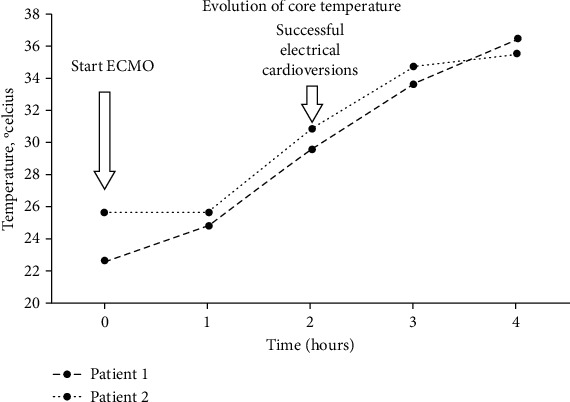
First 4-hour temperature trends in patients 1 and 2.

The second patient, a 30-year-old Caucasian male, was admitted this winter to our Emergency Department with severe hypothermia. Main medical history was also chronic alcohol abuse. The patient was again homeless. Clinical examination revealed profound hypotension (77/42 mmHg), bradycardia (50 bpm), and coma (Glasgow coma scale E2, V2, and M2). Core temperature was 25.3°C. The arterial blood gas analysis showed a severe acidosis with a pH of 6.95, a PaCO_2_ of 26 mmHg, and hyperoxia at 155. Lactic acidosis was present with a first value of 20 mmol/L (<2) as well as a severe anemia with Hb 3 g/dL (13-18). Concentrated red blood cells were administered to maintain Hb above 8.5 g/L. Passive and active warming were started with forced hot air and warmed normal saline perfusions. Cerebral CT excluded any important ischemic stroke or intracerebral hemorrhage. Injected multiple phase abdominal CT excluded internal bleeding. Persistent coma led to intubation and mechanical ventilation with volume control with a tidal volume of 400 mL, 20 times per minute with again at first an FiO_2_ of 100% then after VA ECMO and after correction of hypothermia and anemia; the FiO_2_ was decreased to 45% in order to maintain SpO_2_ at 95%. The total CPR was finally 2.5 hours. Upon Intensive Care Unit arrival, the patient developed a cardiac arrest with ventricular fibrillation. Three defibrillation attempts failed to restore ROSC. No epinephrine was administered. Mechanized CPR was started with a LUCAS® device, and the ECMO team was called. A venoarterial extracorporeal membrane oxygenation system was installed (flow 2 L/min/m^2^, FiO_2_ 100% with femorofemoral approach with a 25 F venous catheter and a 17 F arterial catheter). This permitted to achieve a full flow after 150 minutes. Unfractionated heparin was given with anti-Xa kept between 0.5 and 0.7 IU/mL. At 30°C, the defibrillation attempt was successful as in the first patient. The ECMO was weaned on day 3, and the patient was extubated the next morning. Pulmonary infection with oxacillin-sensitive Staphylococcus aureus was treated for 7 days with flucloxacillin. He was transferred to a normal ward on day 6 to assess the exact nature of the anemia. A full digestive endoscopy including the upper part by esophagogastroduodenoscopy and the lower part with a full colonoscopy did not show any active or sequelae of bleeding. He left our hospital on day 11 with full neurological recovery.  Figure 1 shows the first 24 h trend of temperature in these two patients.

## 3. Discussion

Hypothermia has been divided into 5 groups according to its severity (Table 1) [6]. If correctly treated, cardiac arrests linked to severe hypothermia seem to have good long-term prognosis [7]. Indeed, exceedingly long reanimated cardiac arrest with profound hypothermia has been observed to survive without neurological sequelae [8]. CPR should be withheld in hypothermic patients only if the cause of cardiac arrest is clearly attributable to a lethal injury, fatal illness, or prolonged asphyxia or if the chest is incompressible [9]. Usual signs of death as fixed mydriasis and rigor mortis are not easily applicable to hypothermic patients so deciding to start or withhold CPR is challenging [6]. Identifying no-flow and low-flow times are crucial for prognosticating the success of CPR or eCPR. No-flow should be below 5 minutes and low-flow time beneath 100 minutes. No-flow time is rarely available in out-of-hospital hypothermia-induced cardiac arrest. A surrogate to minimally preserved metabolic activity could be end-tidal CO_2_ (ETCO_2_). An ETCO_2_ above 20 mmHg could be a sign of relatively short no-flow time [10]. In 2013, the American Heart Association recommended ETCO_2_ as the primary physiological metric during CPR when neither an arterial nor a central venous catheter was in place and suggested titrating CPR performance to a goal ETCO_2_ of >20 mmHg [11]. The European Resuscitation Council (ERC) 2015 guidelines suggest using a waveform capnography to assess the quality of CPR but did not provide a specific ETCO_2_ target for resuscitation [12].

**Figure 2:**
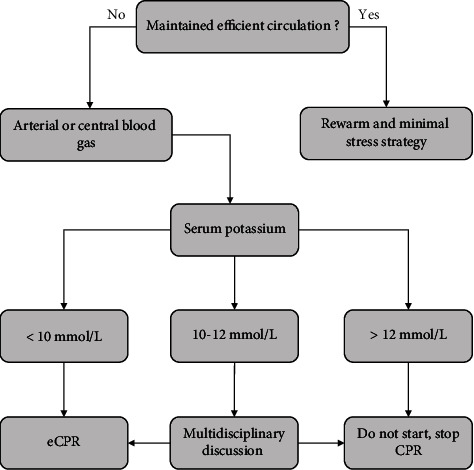
Decisional algorithm to start CPR in patients with severe hypothermia. Multidisciplinary discussion should include patient's age, comorbidities, time of no flow and/or low flow, presence of severe trauma, or prearrest signs of asphyxia.

A literature-derived algorithm is summarized in Figure 2. Evaluation should include core temperature, serum potassium levels (crf. infra), age, comorbidities, and signs of prearrest asphyxia. If CPR is started, epinephrine administration should not be given under 30°C as it may cause myocardial injury [9]. If defibrillation is undertaken, there should be no more than 3 attempts. Rewarming speed is dependent on the technique used. Active external rewarming usually increases core temperature by 0.1 to 3°C per hour, whereas peritoneal lavage, thoracic lavage, or hemodialysis increases core temperature by 1-3, 3, and 2-4°C/h, respectively [13]. ECMO increase core temperature by 4°C per hour [14]. Optimal rewarming speed is ill-defined in the literature. A proposed maximum speed of 4°C/h has been proposed [15] while maintaining a temperature gradient between patient and ECMO of maximum 10°C [16]. An animal study performed on a pig model compared two ECMO flow regimes (1.5 L/min vs. 3 L/min) and two different temperature goals (5 degrees Celsius above body temperature versus 38 degrees Celsius throughout the resuscitation). They observed better cardiac output in the 3 L/min group. Regarding temperature, the gradual warming by 5 degrees Celsius decreased the production of receptor for advanced glycation end products (RAGE) which is involved in the inflammatory cascade [16].

When core temperature is below 30-32°C, another important factor for initiating CPR is serum potassium. Levels above 12 mmol/L are usually associated with poor outcome, and CPR should not be undertaken or should be terminated while under way (Figure 2). Between 10 and 12 mmol/L, eCPR seems the best therapeutic choice and an ECMO team should be called. It is important here to weigh the awaited benefit of such a resource consuming therapy. Age, comorbidities, signs of prearrest asphyxia, or presence of severe trauma before starting an eCPR must be correctly evaluated. This is important because after trauma, patients will need anticoagulation during ECMO with the risk of severe bleeding. Below 10 mmol/L, CPR should be continued until the patient is rewarmed [17]. It is important to have a correct sampling so that the result is reliable as decisions are definite. Below 8-10 mmol/L, CPR and eCPR should be initiated [18] even though a low potassium level does not guarantee survival [17]. Lactate and pH levels have very inconsistent in prognosticating outcome in hypothermia-induced cardiac arrest [19].

In a retrospective study on hypothermic patients accepted for extracorporeal rewarming at the Severe Accidental Hypothermia Center (Cracow), thirteen patients were identified with circulatory instability and were enrolled in the study. The evaluation took into account patients' condition on admission, the course of therapy, and changes in laboratory and hemodynamic parameters. Bad prognostic factors were identified: old age, low blood pressure on admission, low initial and postwarming PaCO_2_ partial pressure, low pH, large base deficit, high serum creatinine, and potassium and lactate levels (marked at least six hours of rewarming) [20].

Recently, authors have studied a tool of hypothermia outcome prediction after extracorporeal life support for hypothermic cardiac arrest patients: the HOPE Score. In a population of 286 patients, 106 survived (37%; 95% CI: 32-43%), most (84%) with a good neurological outcome. The final score developed after a multiple logistic regression model to predict survival included the following variables: age, sex, core temperature at admission, serum potassium level, mechanism of cooling, and cardiopulmonary resuscitation duration. The corresponding area under the receiver operating characteristic curve was 0.895 (95% CI: 0.859-0.931) compared to 0.774 (95% CI: 0.720-0.828) when based on the serum potassium level alone [6].

Recently, a clinical case of a 27-month-old boy who underwent accidental hypothermia to 11.8°C was published. He was resuscitated with prolonged rewarming by extracorporeal membrane oxygenation without significant neurological impairments. This is probably the lowest temperature ever documented, at which a human being has been successfully resuscitated from accidental hypothermia after a long period of circulatory arrest [21].

## 4. Conclusion

These two patients demonstrate the good prognosis of long-lasting cardiac arrest due to severe hypothermia. ECMO is starting to be the treatment of choice after carefully evaluating the patients to ensure the benefit of such a resource consuming device. Evaluation should include core temperature, serum potassium levels, age, comorbidities, signs of prearrest asphyxia, or presence of severe trauma before starting an eCPR. Studies are necessary for providing strong recommendations in the management of severe hypothermia. In out of hospital cardiac arrest due to hypothermia, orientation to an ECMO center should be recommended.

## Figures and Tables

**Table 1:** Groups of hypothermia according to its severity [6].

	Type	Temperature	Symptoms
Group 1	Mild	35–32°C	Conscious, shivering patient
Group 2	Moderate	32–28°C	Impaired consciousness without shivering
Group 3	Severe	28–24°C	Coma, unconsciousness, present vital signs, possibility of rhythm disturbance, bradycardia, wide QRS, and cardiac arrest
Group 4	Very severe	Below 24°C	Cardiac arrest or low flow state
Group 5	Irreversible	Below 13.7°C	Dead patients due to irreversible hypothermia
